# Mr. Martin's Case of Imperforate Anus

**Published:** 1807-10

**Authors:** P. J. Martin

**Affiliations:** Pulborough


### *To the Editors of the Medical and Physical Journal.*

Gentlemen,

On the 2d of December last I was requested by my father to see the child of Mr. L. I learnt, that it was born on the 28th of November; to outward appearance, a well formed, healthy female. On the 30th, it began to shew signs of considerable uneasiness, with some tension of the abdomen, and a constant and increasing inclination to stool. This being attributed by the attendants to a retention of the meconium, a little manna was administered, but immediately rejected, and it refused to take the breast. The next day my father saw it; the symptoms were aggravated, the abdomen more tense, violent straining, occasionally followed by a vomiting of bilious and stercoracious matter. As he did not suspect the real cause, a little Castor oil was ordered, and directions given for the use of the warm bath. These seeming only to exasperate the symptoms, glysters were about to be had recourse to, when it was immediately discovered that the cause of the disorder was an imperforate rectum.

Upon examination, I found that the anus was completely formed, as were the urinary organs, the child having passed its urine naturally from the beginning. On attempting to introduce a bougie, it met with a firm resistance, about half an inch, or a little more, within the anus; and although one of the smallest was used, no opening could be found. The child appeared to be very much exhausted, uttered a faint moaning cry, and was becoming comatose; much tension of the abdomen, and other signs of peritoneal inflammation. It was immediately determined that a puncture should be made.

The canula of a moderate sized trochar was introduced, and held firmly against the resisting part; then passing the stilette (which was triangular at the point, according to the old fashion) along the canula, till the resistance was felt, I pushed it home with a quick motion, so that it should enter just so far as it projected beyond the canula; the stilette being withdrawn, the canula passed easily onward, and thickened meconium was discharged.

As it seemed to impede rather than assist in the evacuation, the canula was soon withdrawn, and nothing ordered but a repetition of the warm bath. All the disagreeable symptoms subsided beyond the most sanguine expectation. The bougie was used within twelve hours after the operation, and its use was persisted in for two or three days days, but afterwards discontinued, at the earnest request of the mother and attendants. The child improved rapidly in appearance, took kindly to the breast, and continued to pass its fæces regularly to the seventh day, when the evacuation suddenly ceased, and all the unpleasant symptoms returned, with vomiting and tension of the abdomen. The resistance to the passage of the bougie was as strong as ever; and although a considerable degree of force was exerted, the opening could not be restored. A few hours were given for the accumulation of fæces behind the stricture, when the operation was performed in the same manner as before, and followed by a sudden and forcible discharge of liquid fæces.

I now took upon me the management of the case, and used the bougie twice a day for the first week, and afterwards once a day, gradually increasing the size of the instrument till little or no appearance of stricture remained. The child was improving the while in health and strength; no medicine was required, except now and then a little mistura cretæ to check the inclination to diarrhœa, and remove acidity. It soon became remarkable for its size and forwardness, beyond what the lady's former children had been; and to my repeated inquiries the answer always was, that every thing went on well; and I began to consider the cure complete.

On the 12th of August, eight months from the time of the operation, I was sent for to see it again, when I found that the rectum had contracted, so as scarcely to admit a common sized urethra bougie. I now also learnt, that since the child had passed a figured stool, it was observed to be flat, like a piece of tape, constantly getting less, and always passed with violent straining. But supposing that all would yet be well, they had neglected to apply for assistance. The use of the bougie was resumed; to this it gradually yielded, and a solid stool now passes with little more than the natural effort, and I shall continue it till the stricture is entirely lost; taking care, for some time to come, to have ocular demonstration of the size and figure of the child's fæces.

The stricture is now nearly an inch within the anus.

A case of malformation of these parts seldom happens so favourable for surgical treatment; and as I have no opportunity at present of consulting any author who gives a detailed description of its treatment, I should be glad to know of any of your Correspondents, who may have had opportunity of observing, how long the disposition to contract is likely to continue. I am, &c.

*Pulborough, Aug.* 31, 1897. P. J. MARTIN.

